# Microfluidics for disease diagnostics based on surface-enhanced raman scattering detection

**DOI:** 10.1186/s40580-024-00424-7

**Published:** 2024-04-30

**Authors:** Xiangdong Yu, Sohyun Park, Sungwoon Lee, Sang-Woo Joo, Jaebum Choo

**Affiliations:** 1 Department of Chemistry, Chung-Ang University, Seoul, 06974 South Korea; 2 Department of Chemistry, Soongsil University, Seoul, 06978 South Korea

**Keywords:** Surface-enhanced Raman scattering, Microfluidics, Lab-on-a-chip, On-chip detection, Biomedical diagnostics

## Abstract

This review reports diverse microfluidic systems utilizing surface-enhanced Raman scattering (SERS) detection for disease diagnosis. Integrating SERS detection technology, providing high-sensitivity detection, and microfluidic technology for manipulating small liquid samples in microdevices has expanded the analytical capabilities previously confined to larger settings. This study explores the principles and uses of various SERS-based microfluidic devices developed over the last two decades. Specifically, we investigate the operational principles of documented SERS-based microfluidic devices, including continuous-flow channels, microarray-embedded microfluidic channels, droplet microfluidic channels, digital droplet channels, and gradient microfluidic channels. We also examine their applications in biomedical diagnostics. In conclusion, we summarize the areas requiring further development to translate these SERS-based microfluidic technologies into practical applications in clinical diagnostics.

## Introduction

Since the introduction of the micro-total analysis system (micro-TAS) by Manz in the early 1990s [1], microfluidic systems have been applied across diverse domains, including biomedical diagnosis, chemical synthesis and analysis, drug discovery, and human healthcare [2, 3]. The global impact of the coronavirus disease of 2019 (COVID-19) has underscored the urgent need for on-site diagnostic systems capable of swiftly and accurately analyzing minute clinical samples [4]. Microfluidic devices are viewed as a pivotal technology for meeting this demand. Microfluidic technology is recognized for its ability to precisely control reactions between proteins or DNAs in miniaturized volumes, and is thus considered indispensable for biomedical diagnosis [5–7]. However, the detection volume in microfluidic channels, typically at the micron or submicron level, is exceedingly small, as measured in nano- or pico-liters. Consequently, developing highly sensitive detection techniques is imperative [8, 9]. In the early developmental stages of microfluidic technology, researchers relied extensively on on-chip optical detection methods such as UV/vis absorption, laser-induced fluorescence, chemiluminescence, and thermal lens microscopy [10–12]. Nevertheless, these approaches have limitations, including poor sensitivity to miniaturized detection volumes and constraints on multiplex detection capability.

Since 2005, a new on-chip detection method that utilizes the aggregation of Au or Ag nanoparticles for surface-enhanced Raman scattering (SERS) detection has emerged for addressing various challenges [13, 14]. This method leverages the localized surface plasmon coupling that occurs in nanogaps between nanoparticles, enabling highly sensitive detection of target molecules through electromagnetic enhancement phenomena [15–18]. In other words, SERS signal amplification can overcome the inherent low sensitivity of on-chip detection using fluorescence and UV/vis absorption. Additionally, SERS peaks have narrower linewidths than fluorescence or UV/vis absorption bands, providing an advantage for the simultaneous detection of multiple samples, a process known as multiplex detection [19, 20]. However, in SERS detection using lasers, the small focal volume may lead to limited reproducibility when measuring numerous detection spots due to the non-uniform distribution of hotspots [21–23]. Fixing laser spots within the microfluidic channel, where target samples flow, and continuously measuring the scattering signal can prospectively enhance the reproducibility owing to ensemble average effects [24–26]. Overall, compared with conventional on-chip techniques, SERS-based microfluidic technology has the potential for significantly improved sensitivity, reproducibility, multiplex detection capability, and other features.

This review discusses the configurations and uses of microfluidic devices designed for SERS detection. These devices incorporate diverse microfluidic channels developed using the on-chip SERS detection technology and microfabrication techniques. Although numerous review articles on SERS-based microfluidic devices have been published over the last two decades, to the best of our knowledge, no documented review has specifically addressed the classification and applications of SERS-based microfluidic chips. In this review, we systematically categorize the reported SERS-based microfluidic chips on the basis of the device characteristics, including continuous-flow channels, microarray-embedded microfluidic channels, droplet microfluidic channels, digital droplet channels, and gradient microfluidic channels. Subsequently, we provide an overview of the distinctive designs and sensor application methods for each channel type. This review offers valuable insights into the design and application of SERS-based microfluidic channels, especially for the effective analysis of characteristic biochemical reactions and biomedical diagnoses.

## Classification of microfluidic devices for SERS detection

### Continuous-flow microfluidic channels

**Fig. 1:**
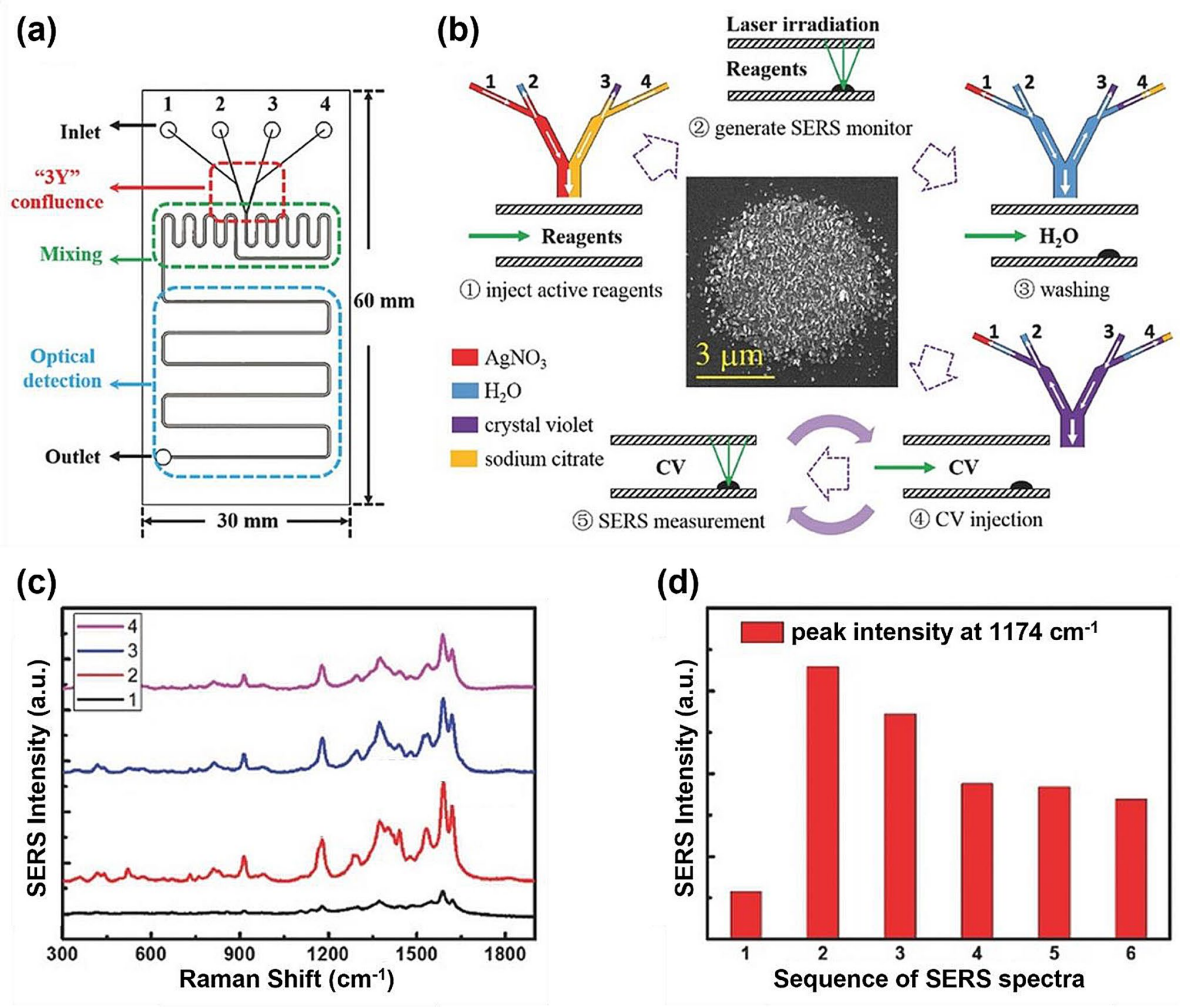
Development of SERS materials and the progression of SERS intensity throughout the measurement sequence. **a** Diagram illustrating the microfluidic chip, featuring three modules designated for injection (red), mixing (green), and optical detection (blue). **b** Sequential steps in the growth of silver nano aggregates through photoinduction and subsequent in situ SERS measurements. Different colored solutions of AgNO_3_, H_2_O, crystal violet, and sodium citrate in the injection module are depicted, with the flow in the microchannel indicated by white arrows. **c** Collection of SERS spectra utilizing a 532 nm laser with a power of 130 µW. The integration time for each spectrum is 10 s. **d** Raman intensity histogram of CV at 1174 cm^− 1^ in the sequence of SERS measurements. Reprinted with permission from [36]. Copyright 2017 Wiley-VCH Verlag GmbH & Co

Reproducibility is one of the most challenging issues in the quantitative analysis of target molecules using SERS technology [27, 28]. In other words, controlling the degree of aggregation when forming nanogaps using gold nanoparticles is difficult [29]. Achieving a uniform distribution of target molecules on the substrate surface when measuring Raman signals using nano-substrates is also a challenge [30]. The use of SERS-based microfluidic technologies can address these issues. Controlling reactions in a continuous-flow regime within microfluidic channels maintains homogeneous mixing conditions, enabling sensitive and reproducible detection [31, 32]. The early days of SERS-based microfluidics, introduced in the mid-1990s, initially focused on developing micromixers capable of effectively mixing Au/Ag nanoparticles with the target molecules [33–35]. Yan et al. developed a microfluidic system capable of detecting single molecules through two stages of photoinduced reduction (Fig. 1) [36]. Using a focused laser beam at the inlet of the microfluidic channel, they generated silver nanoparticles (AgNPs) through the first photo-induced reaction. Continuous scanning of the same laser beam over the residual reactants and AgNPs induced a second reduction reaction, effectively positioning the residual reactants in the nanogaps of the AgNP aggregates to form strong hotspots. During this process, the analyte is removed through photoreduction and photodegradation, virtually eliminating the memory effect caused by the residual reactants. By employing SERS-based microfluidic channels and laser-induced photoreactions, the authors successfully detected substances such as crystal violet, rhodamine 6G, methylene blue, hemoglobin, and 5-fluorouracil at concentrations as low as 10^− 13^ M.

**Fig. 2:**
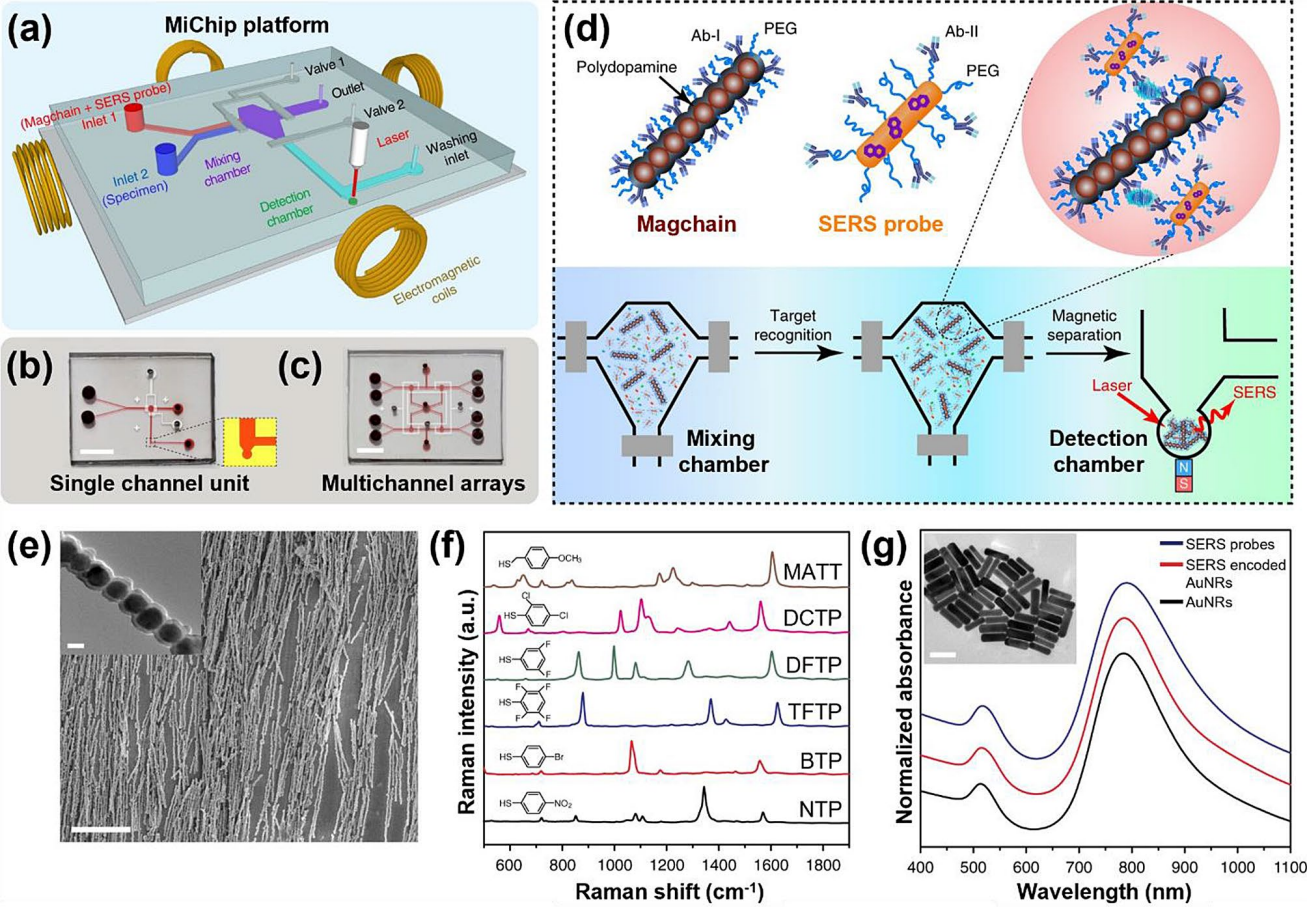
Design of the MiChip integrated with magnetic nano-chains. **a** Schematic of MiChip. **b** Photographs of MiChip showcasing a single-channel unit and multichannel arrays. **c** To enhance visibility, the microchannel in the images is filled with a red dye, with a scale bar of 0.5 cm. **d** MiChip assay for biomarker detection. The specimen, antibody-conjugated magnetic nano-chains (Magchain), and SERS-encoded probes are combined in the mixing chamber. The antibodies on the Magchains and the SERS probes recognize the targets of interest in the specimen, forming sandwich immune complexes. These complexes are subsequently isolated into the detection chamber for Raman detection. **e** A close-up SEM image of the magnetic nanochains, with a scale bar of 20 μm, while the inset shows a TEM image of the nanochain with a scale bar of 200 nm. **f** SERS spectra of six representative SERS-encoded gold nanorods (AuNRs), including 4-nitrothiophenol (NTP), 4-bromothiophenol (BTP), 2,3,5,6-tetrafluorothiophenol (TFTP), 3,5-difluorothiophenol (DFTP), 2,4-dichlorothiophenol (DCTP), and 4-methoxy-α-toluenethiol (MATT). **g** UV–vis spectra of original AuNRs, SERS-encoded AuNRs, and antibody-conjugated SERS probes. The inset includes a TEM image of the AuNRs with a scale bar of 100 nm. Reprinted with permission from [40]. Copyright 2018 Springer Nature

With the development of effective immunoassays for disease diagnosis in the mid-2000s, microfluidic chips have continued to be developed [37–39]. A notable example is the microfluidic biochip (MiChip) integrated with magnetic nano-chains created by Xiong et al., which enabled rapid liquid mixing and simultaneous bio separation for the ultrasensitive detection of cancer protein biomarkers and bacteria (Fig. 2) [40]. As depicted in the figure, a coil capable of generating a magnetic field was embedded in the MiChip. Capture antibody-conjugated Magchains operated with a magnetic stirrer were utilized, and detection antibody-conjugated SERS-encoded probes were mixed within the chamber, effectively generating magnetic immunocomplexes. Subsequently, the complexes were magnetically separated into a detection chamber, followed by SERS analysis.

**Fig. 3:**
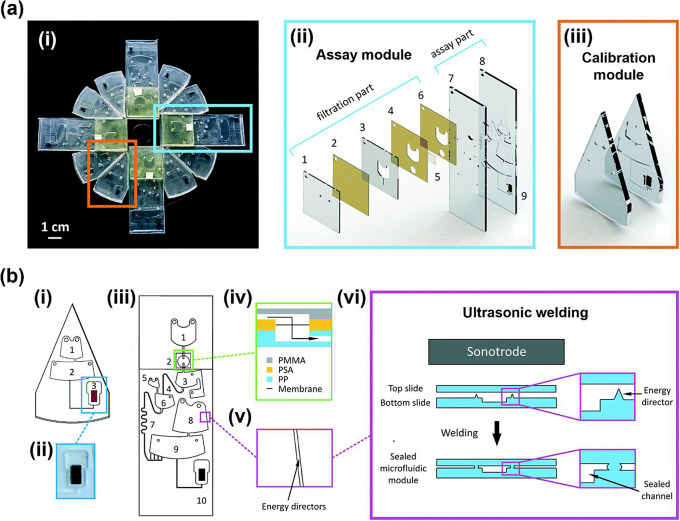
**a** (i) Development of a Lab-on-a-Disc (LoD) system for liquid-liquid extraction and identification of bacterial metabolites. The LoD device comprises 12 modules arranged on a PMMA disc. (ii) Diagram illustrating the components of the assay module, featuring a filtration parts. (1–6) with a cellulose acetate membrane (5) and an assay segment (7–9) incorporating an embedded SERS chip. (iii) Schematic representation of the calibration module with the integrated SERS chip, presented in an exploded view. **b** (i) The fluidic configuration of the calibration module, (ii) A SERS chip is affixed using PSA tape. (iii) The microfluidic design of the assay module (iv) Schematics of the filtration chamber. The incorporation of the cellulose acetate membrane between PSA layers, along with the indicated flow direction. (v) A representation of energy directors positioned at the edge of a microfluidic chamber. (vi) A schematic representation of the working principle of ultrasonic welding. Reprinted with permission from [41]. Copyright 2018 Royal Society of Chemistry

Morelli et al. developed an automated centrifugal microfluidic device comprising eight calibration modules with integrated sample pretreatment functions, and four assay modules with SERS detection capabilities (Fig. 3) [41]. They successfully utilized the fabricated microfluidic channels to detect a secondary bacterial metabolite (*p*-coumaric acid) in bacterial supernatant, with high sensitivity. The developed lab-on-a-disk device was assessed as a new tool for the real-time and high-throughput screening of various bacteria.

### Microarray-embedded fluidic channels

**Fig. 4:**
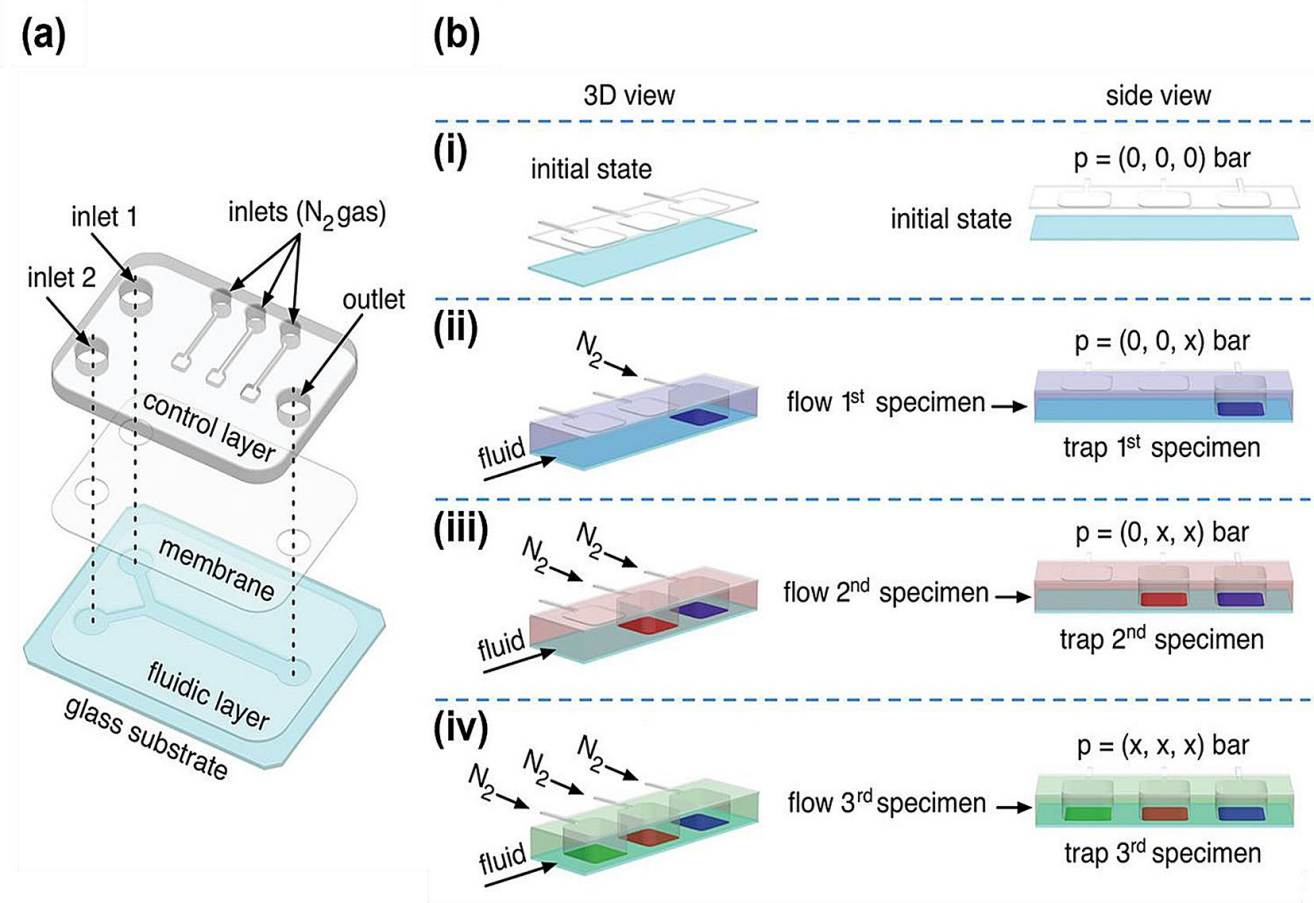
**a** Detailed diagram of the double-layer microfluidic device employed in the experiments. **b** Diagrams depicting the precise positioning of three specimens (blue, red, and green) within the fluidic layer of the microfluidic device described in Panels (i–iv) present the step-by-step process for capturing the three specimens. The symbol “x” denotes pneumatic actuation with nitrogen gas. Reprinted with permission from [45]. Copyright 2020 Wiley-VCH Verlag GmbH & Co

Most microfluidic devices are limited to the analysis of only a single target. Microarray-embedded microfluidic devices composed of multiplex nanostructured components that simultaneously measure multiple targets have been developed to overcome this limitation [42–44]. Sevim et al. developed a device with numerous SERS substrates embedded within a single microfluidic channel for detecting various analytes simultaneously on a single microfluidic chip (Fig. 4) [45]. This design employs microfluidic spatial control to localize multiple target gas samples on multiple SERS substrates.

**Fig. 5:**
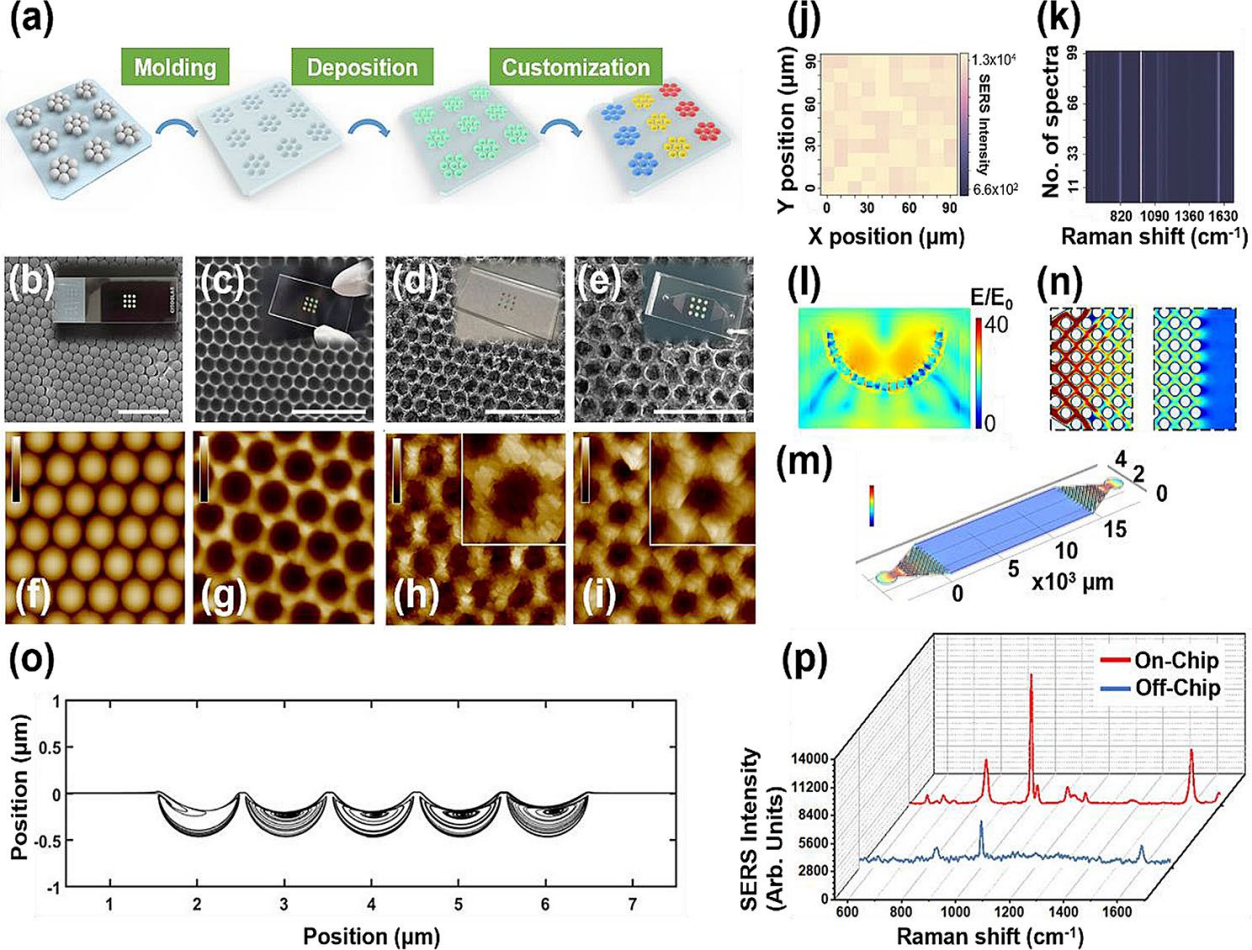
Creation and analysis of the gas sensor. **a** The schematic diagram for the sensor’s preparation. A nine-dot polystyrene (PS) pattern is initially transferred from a monolayer of PS microspheres to a glass slide using the tape-assisted transfer technique, Subsequently, a PDMS duplicate is obtained through molding. Bimetallic nanocubes are then deposited via a self-assembly approach. Finally, three distinct detection units—UA, UB, and UC—are achieved through customized modification. SEM images of **b** the patterned PS mold, **c** the negative PDMS duplicate, **d** the substrate after nanocube deposition, and **e** the substrate following Ti_3_C_2_T_x_ Mxene deposition. All scale bars are set at 5 μm. Inserted images include corresponding photographs. (**f − i**) AFM images corresponding to each step in the preparation process of the array. Inserted images detail the characterization of single micropits, with the color bar indicating height ranging from 0 to 500 nm. **j** Benzaldehyde mapping results based on SERS intensity at 1004 cm^− 1^. **k** stacked image of SERS spectra of benzaldehyde at random regions. **l** Numerical electromagnetic field simulation in a single pit with bimetallic nanocubes using an excitation wavelength of 632.8 nm. **m** simulated gas flow field in the microchannel, with a color bar ranging from 0 to 40 mm/s. **n** Magnified top view of the channel near the entrance and exit of the premixer. **o** Streamlines near the micropits. **p** SERS spectra of benzaldehyde obtained on-chip and off-chip. Reprinted with permission from [46]. Copyright 2022 American Chemical Society

Yang et al. developed a SERS-based microfluidic channel by embedding a microarray of nanoparticles in a microfluidic channel, enabling the simultaneous measurement of various volatile organic compounds (VOCs), as shown in Fig. 5 [46]. Sensitive and simultaneous measurement of various VOCs adsorbed on SERS substrates through physisorption or chemisorption mechanisms was possible. This technology can be directly applied to indoor air pollution monitoring or exhaled-breath-based diagnoses.

### Microdroplet channels

The microfluidic platform operates via two flow regimes: continuous and segmented (droplets). While the continuous flow regime is advantageous for easily creating homogeneous mixing conditions that facilitate quantitative analysis of the generated products, the deposition of nanoparticle aggregates (memory effects) on the channel surface is a disadvantage when using SERS detection technology with AuNPs or AgNPs [47]. This memory effect leads to decreased sensitivity and reproducibility [36, 48]. A microdroplet system using two-phase liquid-flow segmented flow was developed to address the memory effect issue [49–51]. Using this system, monodisperse, nanoliter-sized liquid droplets can be generated in a high-throughput manner within an immiscible carrier fluid. Compared to the single-phase flow, the two-phase segmented flow offers enhanced reproducibility by measuring the Raman signals of rapidly moving droplets, without memory effects.

Park et al. developed a groundbreaking microdroplet sensor that significantly enhanced the sensitivity and reproducibility of SARS-CoV-2 detection [52]. Diagnosis of SARS-CoV-2 has traditionally involved lateral flow assay strips for detecting nucleocapsid protein biomarkers. However, owing to sensitivity limitations, false-negative diagnostic ratios have increased. Additionally, the RT-PCR method for detecting viral RNA has the drawbacks of lengthy diagnostic times and difficulty in on-site diagnosis. To overcome these challenges, we developed a microdroplet sensor for SARS-CoV-2 detection with dramatically improved sensitivity and reproducibility.

**Fig. 6:**
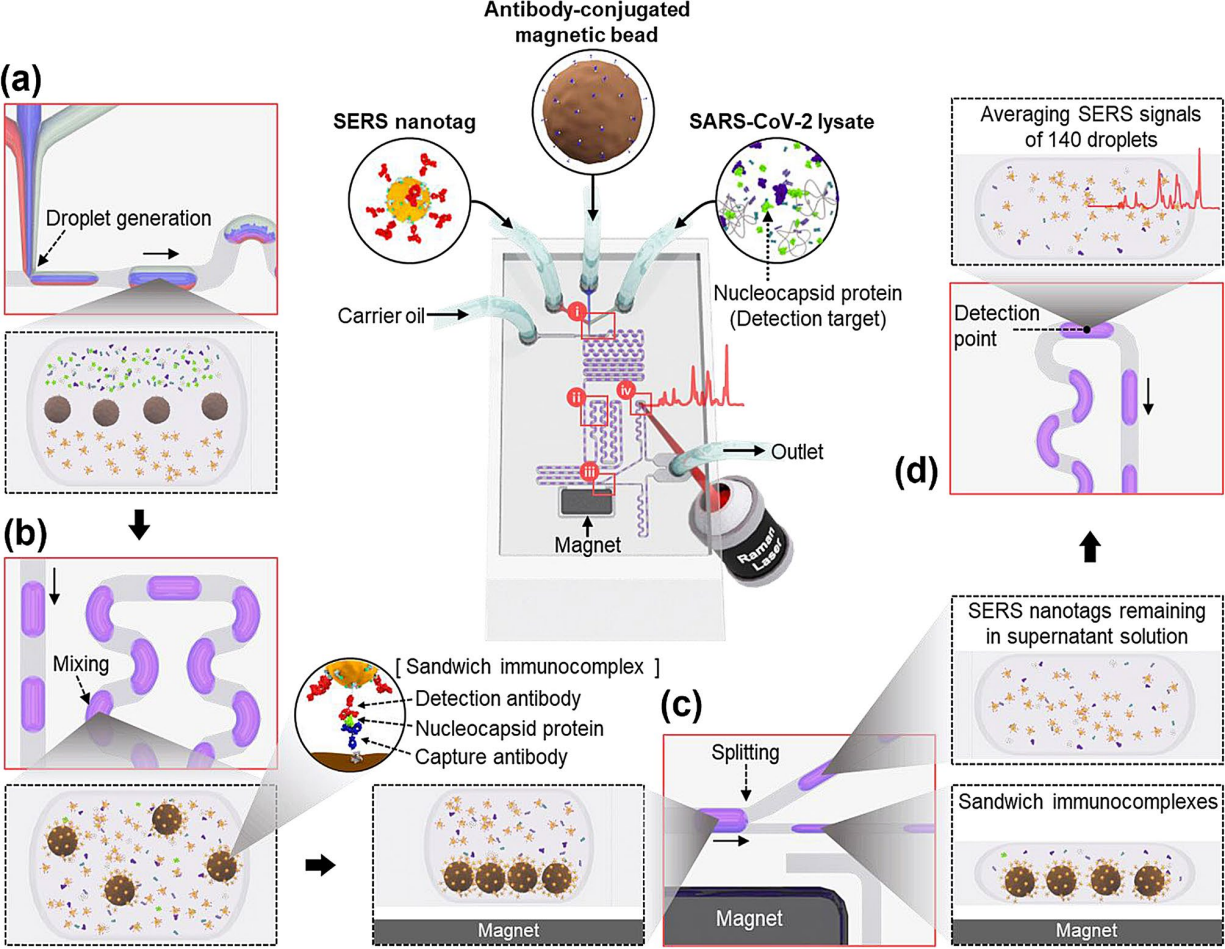
Illustrative diagram of the microdroplet channel designed for SARS-CoV-2 detection. The microfluidic channel comprises four segments: **a** generation of droplets, **b** mixing of droplets, **c** splitting of droplets, and **d** measurement of optical signals. Reprinted with permission from [52]. Copyright 2022 Elsevier

Figure 6 illustrates the structure of the microdroplet sensor used for detecting SARS-CoV-2. The channel is composed of four compartments: (i) droplet generation, (ii) droplet mixing, (iii) droplet splitting, and (iv) SERS detection. By simultaneously injecting the SARS-CoV-2 lysate, capturing the antibody-conjugated magnetic beads, and detecting the antibody-conjugated SERS nanotags in the three inlets of the microfluidic channel, an immunoreaction occurs between the three components as they pass through the winding channel, forming sandwich magnetic immunocomplexes. The generated magnetic immunocomplexes pass through splitting channels containing embedded magnetic bars, separating them from the supernatant solution and removing unreacted SERS nanotags and non-binding species. Finally, the optical signal of the remaining SERS nanotags in the supernatant solution is measured, allowing quantification of the SARS-CoV-2 lysate based on the concentration-dependent SERS signal. When using this SERS-based microdroplet sensor for SARS-CoV-2 detection, the authors achieved a limit of detection (LOD) of 0.22 PFU/mL with a coefficient of variation (CV) of 1.79%, and detection was possible within 10 min.

**Fig. 7:**
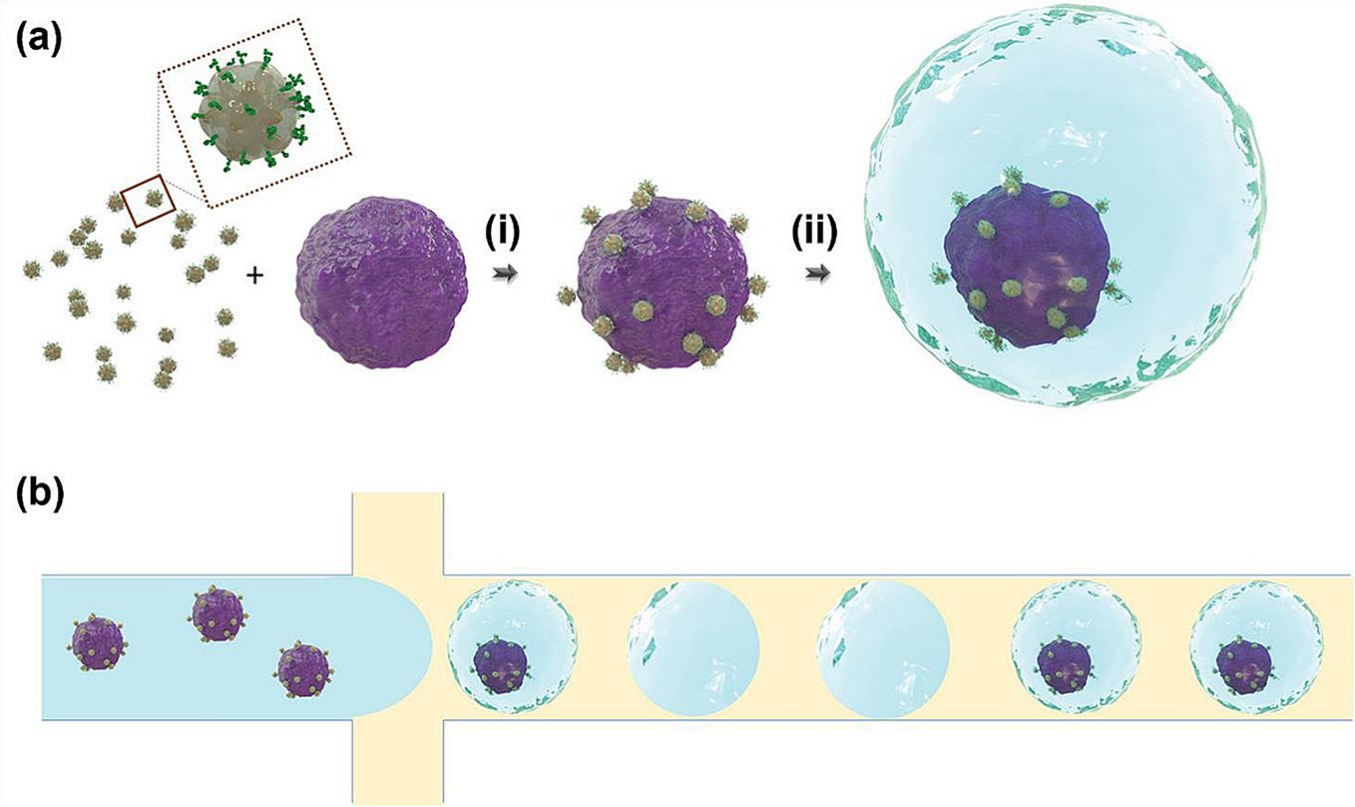
**a** Schematic illustration of cancer cell labeling (depicted as purple spheres) involving (i) SERS tags and their confinement within microdroplets for (ii) single-cell analysis. **b** Diagram outlining the encapsulation procedure, where SERS-tagged cells travel through a central microfluidic channel (colored blue), and the channel’s contents are segmented into droplets by two transverse streams of fluorinated oil and surfactant (colored yellow). The proportions of elements in this figure are not accurately represented to scale. Reprinted with permission from [53]. Copyright 2023 Wiley-VCH Verlag GmbH & Co

Oliveria et al. developed a SERS-based microfluidic sensor capable of multiplex phenotype analysis of single cancer cells (Fig. 7) [53]. Cancer cells labeled with different SERS nanotags capable of recognizing membrane proteins were individually encapsulated in microdroplets. Subsequently, the microdroplets were imaged using SERS spectroscopy. This SERS-based microdroplet sensor represents a novel technique that allows oncologists to rapidly discern the phenotype of cancer cells in a high-throughput manner, thereby enabling rapid therapeutic decision-making.

**Fig. 8:**
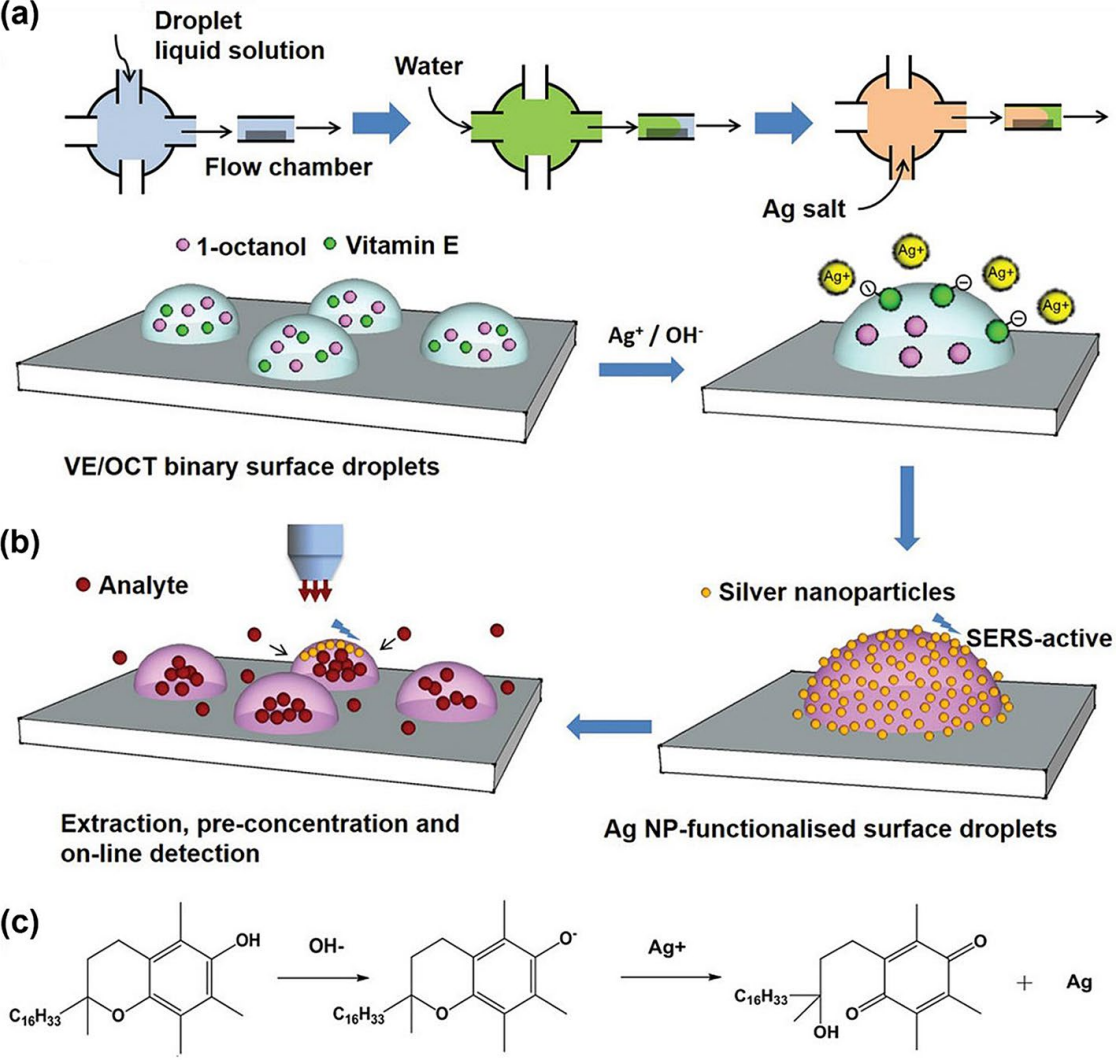
**a** Diagram illustrating the programmed flow sequence to automate the formation of functionalized surface droplets. **b** Illustration depicting the extraction, concentration, and in situ detection relying on surface droplets. **c** The reaction scheme depicting the synthesis of silver nanoparticles through the reaction of vitamin E with AgNO_3_. Reprinted with permission from [54]. Copyright 2019 Wiley-VCH Verlag GmbH & Co

Li et al. developed a femtoliter surface oil droplet-based microfluidic channel for the trace analysis of hydrophobic compounds in aqueous solutions (Fig. 8) [54]. In this system, femtoliter droplets are formed on the surface of a solid substrate within a simple microfluidic chamber, enabling the sequential extraction and concentration of target molecules. The AgNPs adhere to the droplet surface, forming SERS-active hotspots. This droplet system offers advantages such as a large surface-to-volume ratio, tunable chemical composition of the droplets, and long-term stability. This droplet system can prospectively be utilized for the sensitive analysis of various chemical compounds.

### Digital microfluidics

**Fig. 9:**
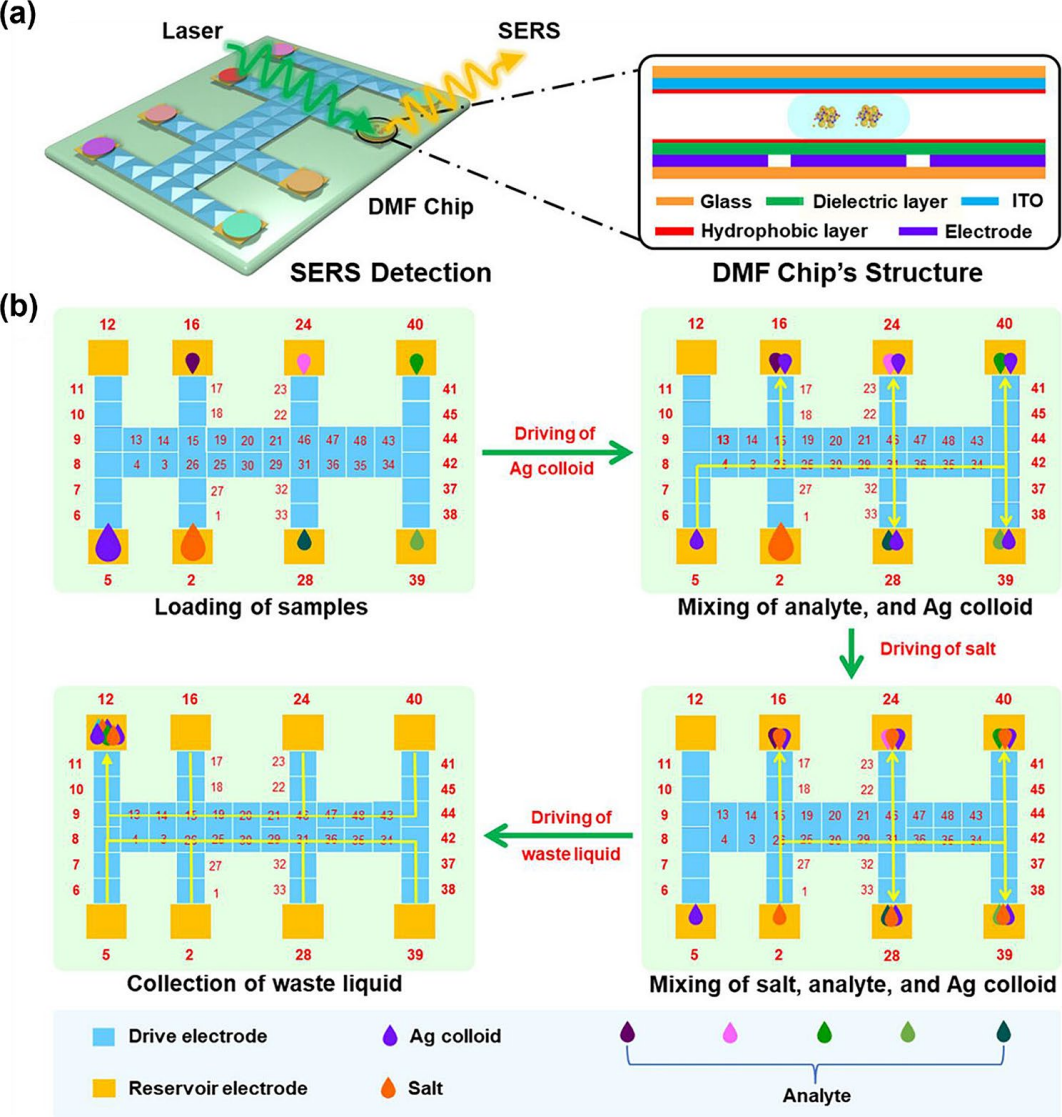
Schematic illustration of the SERS-DMF platform. **a** The structure of the DMF chip. **b** The operational principle of the SERS-DMF platform emphasizes its capability for detecting high-throughput explosives. Reprinted with permission from [58]. Copyright 2023 American Chemical Society

To address the challenges associated with conventional microfluidic channels requiring external pumps and valves for flow control, which complicate downsizing and pose issues such as channel dead volume and cross-contamination, a digital microfluidic (DMF) chip driven by dielectric electrowetting was developed [55–57]. This chip operates without pumps or valves instead of applying a potential to an electrode array on the substrate to enable droplet transport, merging, splitting, and distribution. Liu et al. developed a SERS-based DMF chip for the automated and highly sensitive detection of explosives in a high-throughput manner (Fig. 9) [58]. A DMF chip with 40 drives and eight storage electrodes was fabricated to implement a high-throughput process. Different concentrations of the target molecules, silver nanoparticles (AgNPs), and salts were loaded onto the DMF chip. Subsequently, droplet merging, incubation, and detection processes were automated using the SERS-DMF platform. During these processes, hotspots inducing SERS effects were formed by the AgNPs, facilitated by the use of salts at varying concentrations. The authors utilized this SERS-DMF platform to measure the concentrations of two types of explosives, trinitrotoluene and 3-nitro-1,2,4-triazol-5-one, achieving LODs of 10^− 7^ M and 10^− 8^ M, respectively, for each explosive.

**Fig. 10:**
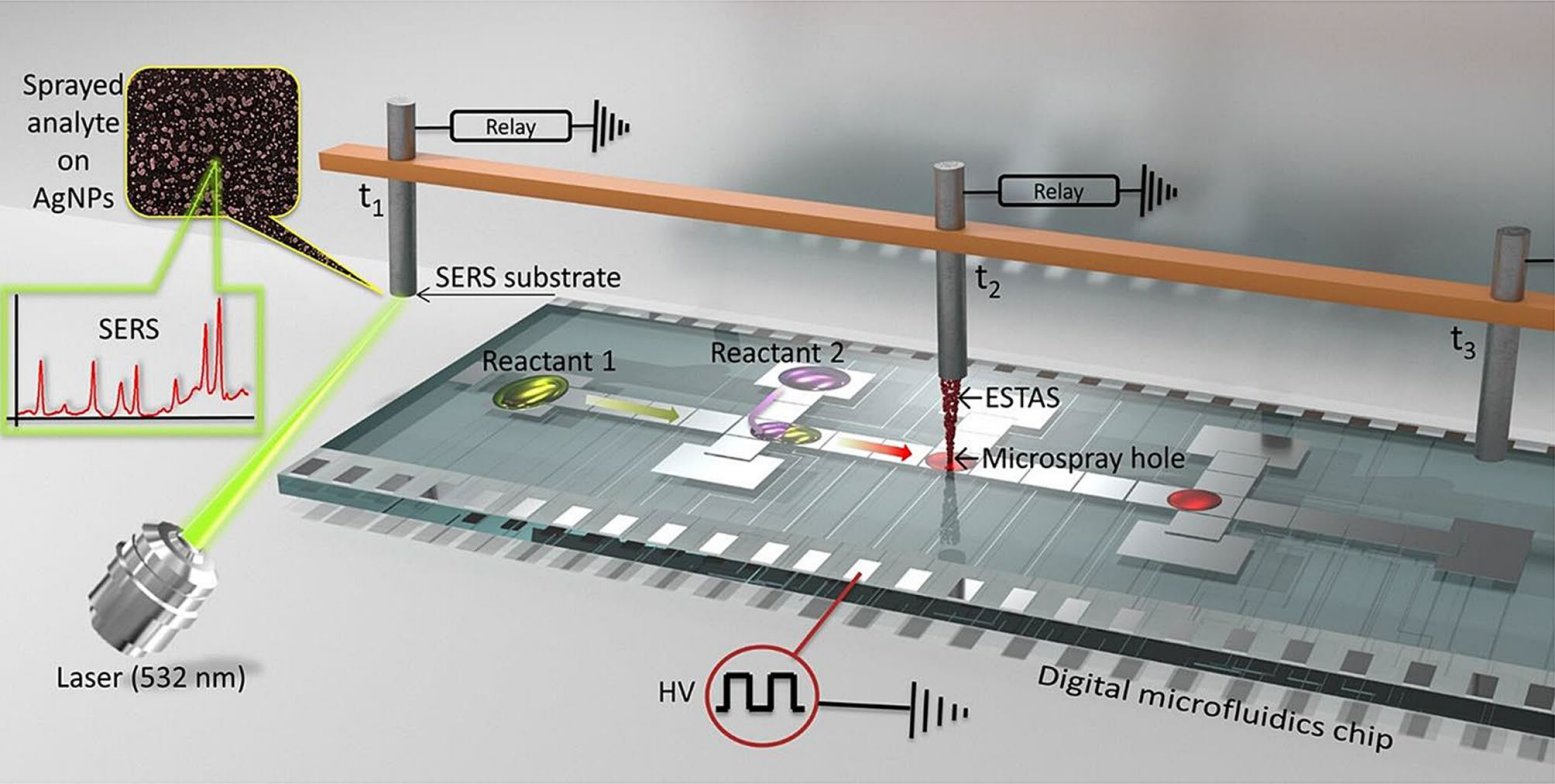
Illustration of the ESTAS-SERS process within a digital microfluidic chip featuring an integrated microspray hole. The initial components are combined on the chip and conveyed beneath the microspray hole (µSH). The reaction solution is initially periodically sampled through electrostatic spray (ESTAS) to deposit the analyte onto the SERS substrate. Subsequently, the SERS substrate containing dried analytes is positioned in the Raman microscope’s beam path for SERS measurement. Concurrently, a fresh sample is activated beneath the µSH. Reprinted with permission from [59]. Copyright 2023 American Chemical Society

Das et al. developed a SERS-DMF platform that utilizes electrostatic spraying (ESTAS) to move a target sample from microdroplets formed on a DMF chip to an external SERS substrate for measurement (Fig. 10) [59]. This technology is highly effective for monitoring the organic reactions in DMF devices. Moreover, the micro spray-hole chip system with ESTAS allowed SERS detection within the chip without colloidal nanoparticles.

### Gradient microfluidic channels

Several technical problems arise when performing bioassays using gold-patterned microarray chips [60–62]. First, achieving a uniform distribution of the target biomarkers on the gold well is challenging because of the limited control of the homogenous immobilization and washing of the target molecules when utilizing a micropipette. Second, because all biomolecules are exposed to air before being fixed on the surface of the gold well, exposure of the biomolecules to air may reduce their biological activity. Finally, repeating the washing steps to remove non-specifically bound species is inconvenient. Additionally, for analyzing various concentrations of target molecules, stock solutions must be prepared, and assays must be performed for each concentration after serial dilution. Therefore, new assays that can overcome the drawbacks of manually controlled and time-consuming assays are required. This can be achieved using programmable and fully automatic gradient microfluidic channels.

**Fig. 11:**
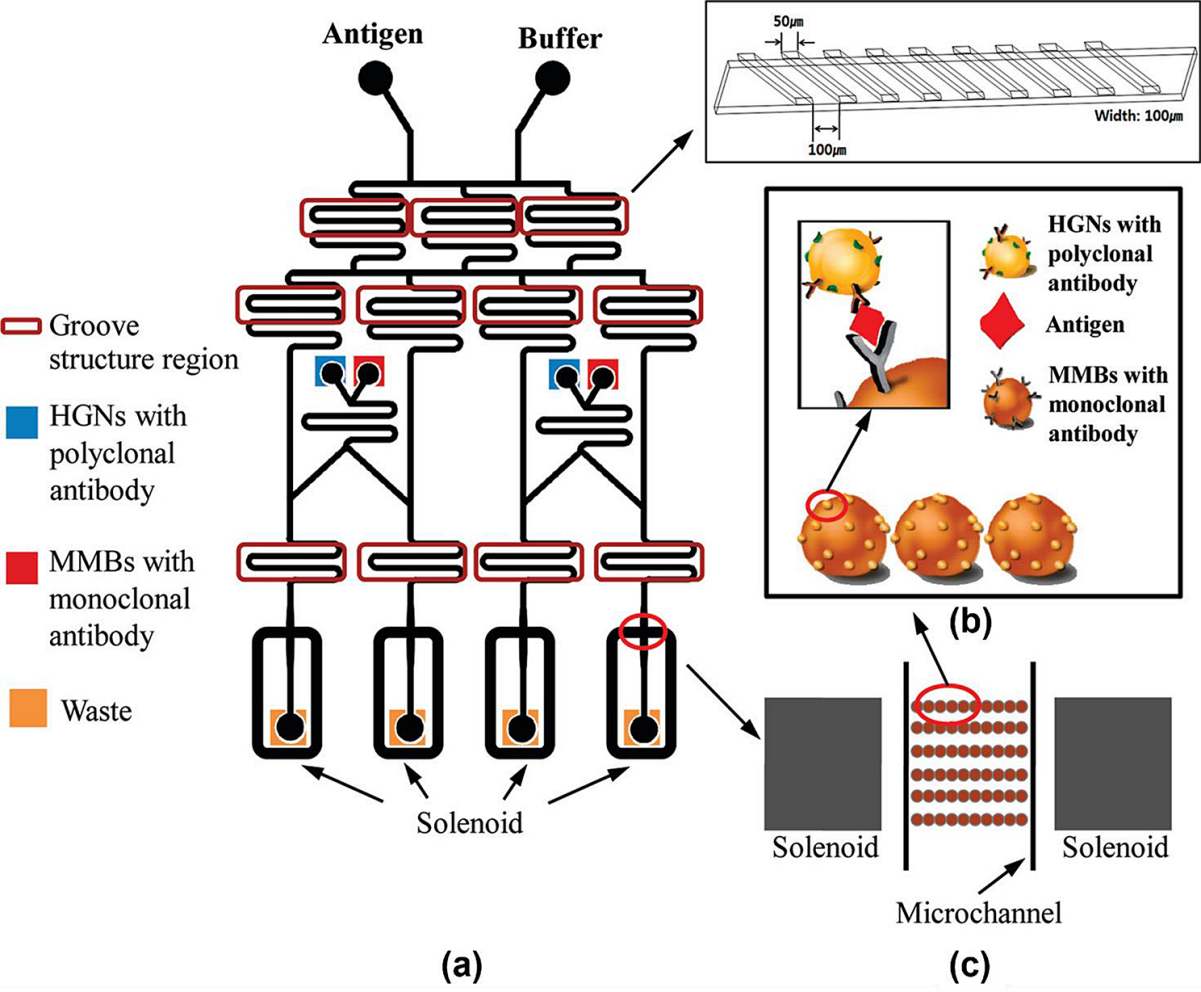
**a** Arrangement of an optofluidic sensor utilizing surface-enhanced Raman scattering (SERS) with integrated solenoids. **b** Creation of a sandwich immunocomplex through the interaction between hollow gold nanospheres (HGNs) and magnetic beads. **c** Capture of the formed sandwich immunocomplexes within a microfluidic channel. Reprinted with permission from [63]. Copyright 2010 American Chemical Society

Chon et al. developed a gradient microfluidic channel that could automatically obtain serial dilutions of target molecules, as shown in Fig. 11 [63]. A chip embedded with solenoid magnets was fabricated in a microfluidic channel capable of generating gradient flows at various concentrations. The chip consisted of three compartments. In the first compartment, the gradient channel structure automatically generated a series of antigen solutions of different concentrations. In the second compartment, antibody-conjugated magnetic beads and detection antibody-conjugated hollow gold nanospheres (SERS nanotags) were injected to form magnetic immunocomplexes under flow conditions with different concentrations of the target antigens generated in the first compartment. A groove-shaped mixer was incorporated to enhance the mixing efficiency in the channels of the first two compartments. In the last compartment, two solenoids were placed on either side of the channel to effectively trap the generated magnetic immunocomplexes. The SERS signal of the captured magnetic immunocomplexes on the channel wall was measured, enabling the quantitative analysis of four different concentrations of antigens. Overall, the SERS-based gradient microfluidic channel enabled automatic and highly reproducible detection of the four antigens at different concentrations.

**Fig. 12:**
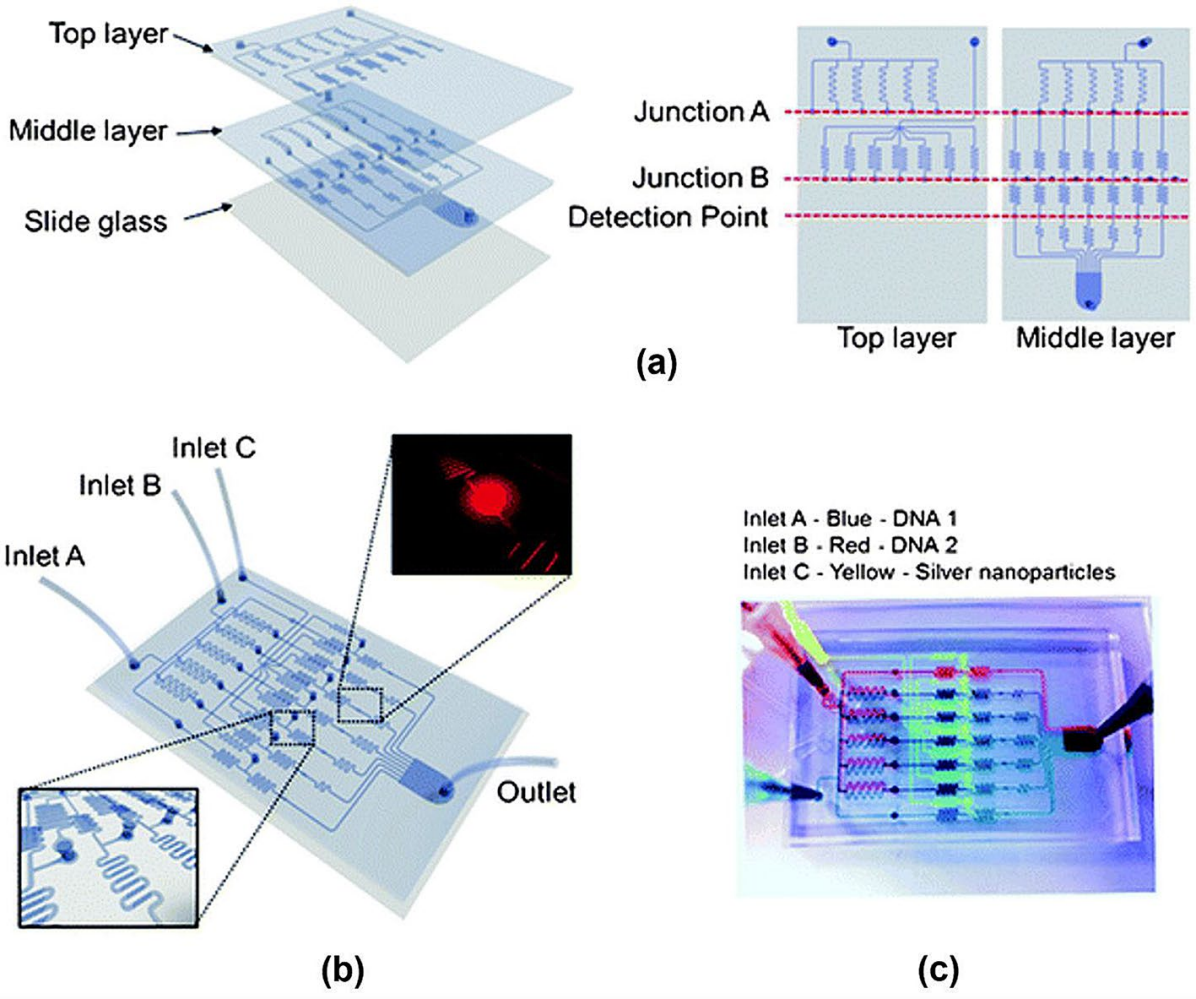
Design of a micronetwork gradient channel based on SERS for quantifying two DNA oligomer mixtures. **a** Upper layer: PDMS panel for creating different concentrations of DNA 1 and uniformly dispersing silver nanoparticles; middle layer: PDMS panel for generating various concentrations of DNA 2, along with groove channels for effective mixing; lower layer: slide glass for PDMS bonding. **b** Integrated three-layer micro-network gradient channel, and **c** photograph of the channel filled with differently colored inks (blue, red, and yellow) injected through the three inlets Reprinted with permission from [64]. Copyright 2012 Royal Society of Chemistry

Choi et al. developed a SERS-based micro network gradient microfluidic platform for two types of DNA oligomer mixtures related to breast cancer (Fig. 12) [64]. The authors designed a microfluidic circuit using an electric-hydraulic analogy to create an on-demand concentration gradient. This microfluidic platform enabled the measurement of SERS signals from duplex DNA oligomer mixtures in different ratios under flow conditions. This microfluidic sensor can automatically generate highly accurate concentration ratios of two DNAs through gradient flow control without tedious manual mixing. Moreover, the assay time is less than 10 min. The Antimicrobial Susceptibility Test (AST) is a pivotal procedure for the prompt identification of suitable antibiotics for patients with bacterial infections. Determining the minimum inhibitory concentration of a specific antibiotic for AST is of paramount significance [65]. Assessing bacterial responses to diverse antibiotic concentrations and determining the optimal antibiotic concentration are essential effectively prescribing antibiotics. However, preparing a series of antibiotic concentrations is tedious and labor-intensive.

**Fig. 13:**
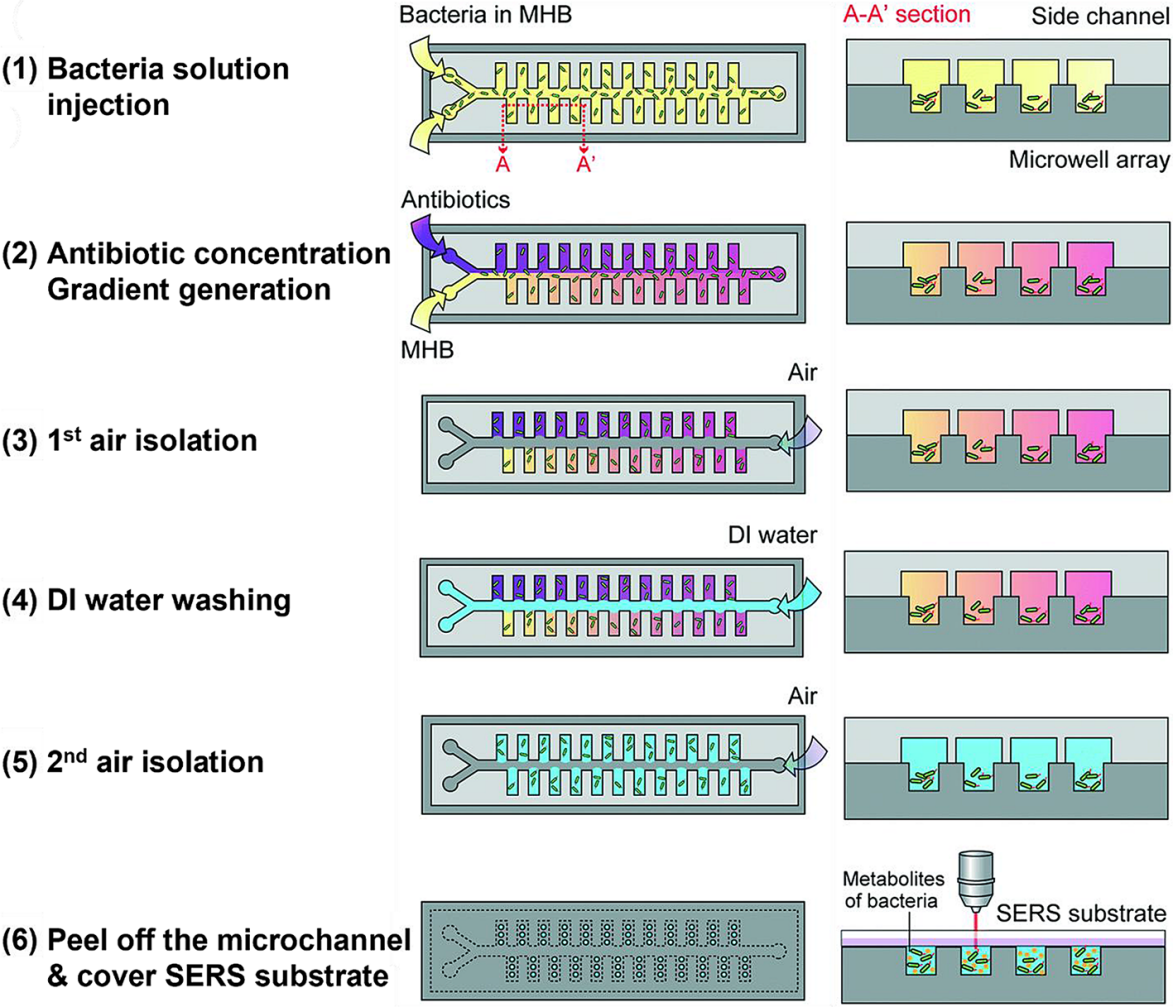
The multiplex SERS-AST procedure performed using the ACGM device. The steps involved are as follows: (1) Injecting a bacterial solution into both inlets, (2) Generating an antibiotic concentration gradient by separately loading the antibiotic in the MHB (purple) and pure MHB (yellow) into two inlets at 0.4 µL min^− 1^, (3) Isolating the first air to segregate each side channel, conducting bacterial treatment at various antibiotic concentrations for 3 h, (4) Washing with DI water to eliminate the bacterial broth within the side channel and microwells, (5) Isolating the second air to separate each side channel, allowing bacteria to secrete metabolites in a nutrient-deficient environment, and (6) Replacing the top microchannel layer with a SERS substrate for SERS measurement. Reprinted with permission from [66]. Copyright 2022 Royal Society of Chemistry Reprinted with permission from [64]. Copyright 2012 Royal Society of Chemistry

To address this challenge, Lin et al. devised a SERS-based antibiotic concentration gradient microfluidic device for multiplex antimicrobial susceptibility tests, as illustrated in Fig. 13 [66]. The main channel facilitates the flow of the antibiotic solution and pure medium to generate antibiotic concentration gradients. These gradients subsequently diffuse into the side channels through a laminar flow regime and are distributed to the bacteria in each microwell. Every stage of the AST, including bacterial loading, antibiotic gradient generation, washing, and bacterial growth with an antibiotic, can be executed on a single chip. Alterations in the bacterial cells loaded onto SERS-active substrates within each microwell are monitored by SERS detection following antibiotic treatment. The SERS-based AST method requires a smaller sample volume of approximately 20 µL and yields results within 5 h compared to traditional AST methods, offering efficiency advantages. In this review, various types of SERS-based microfluidic chips developed so far have been introduced. We compared their key features, advantages, and limitations in Table 1.

**Table 1:** Classification of microfluidic chips for SERS detection

Microfluidic channel	Key features	Limitations	Ref.
Continuous-flow channels	Easily creating homogeneous mixing conditions	Memory effects and limits in reproducibility	[36, 40, 41]
Microarray-embedded channels	Favorable for multiplex detection	Challenging in chip fabrication	[45, 46]
Microdroplet channels	Enhanced reproducibility	The need for a two-phase segmented flow	[52–54]
Digital microfluidics	No need for an external pump and valve Driven by dielectric electrowetting	The limitation of automation due to not being operated under flowing conditions	[58, 59]
Gradient microfluidic channels	Simultaneous assays on samples with various concentrations	Complicated chip structure and challenging fabrication process	[63, 64, 66]

## Conclusion and outlook

This comprehensive review explores the diverse microfluidic devices that utilize SERS for disease diagnosis. Microfluidic technology, which offers a synergistic combination of high sensitivity and precise sample control using nano-liter volumes, is anticipated to find active applications in point-of-care (POC) diagnosis, particularly for swift and accurate assessments of infectious disease scenarios. In the realm of in vitro diagnostics, where diseases are identified by analyzing human fluids such as blood, urine, and nasal discharge, chemiluminescence or fluorescence detection based on light absorption‒emission processes has traditionally dominated. However, these methods often have low sensitivity or present logistical challenges for onsite diagnosis. Consequently, the SERS detection method has emerged as a solution for overcoming these limitations and holds substantial promise as a next-generation POC diagnostic technology.

The effective implementation of this SERS microfluidic technique requires several technological advancements. First, there is a demand for the miniaturization of Raman spectrophotometers. Although numerous portable Raman spectrophotometers have been commercialized, the continuous development of user-friendly, handheld, and touch-panel-enabled Raman spectrophotometers is essential for seamless application in on-site diagnostics. For instance, recently Joung et al. developed a portable Raman reader capable of quantitatively measuring the Raman signal of the test and control lines of a SERS-based lateral flow assay (LFA) strip. This SERS-based POC assay system has approximately 100 times higher sensitivity than commercialized strips that determine infection status through color changes. When utilized for COVID-19 diagnosis, it can significantly reduce the false-negative diagnostic rate [67, 68].

Second, miniaturizing syringe pumps and developing techniques for precise flow control within microfluidic channels without pumps is imperative. Despite the potential for miniaturization of microfluidic devices, challenges persist in downsizing the pumps required for flow control, which hinders the construction of POC systems. Third, establishing a Raman spectral database for amassed clinical samples and integrating machine learning (ML) technology based on this spectral database are crucial for enhancing diagnostic accuracy. Finally, active translational research is indispensable for translating the developed highly sensitive measurement technologies from the laboratory to real clinical settings. Rapid translation necessitates close collaboration among Raman spectroscopists, engineers for developing portable spectrometer systems, software engineers for crafting spectroscopic drivers and ML algorithms, and medical professionals to oversee clinical validation and field usage. To effectively leverage these technologies in clinical settings, collaborative and targeted research efforts are imperative.
